# Biological and Nutritional Properties of Palm Oil and Palmitic Acid: Effects on Health

**DOI:** 10.3390/molecules200917339

**Published:** 2015-09-18

**Authors:** Annamaria Mancini, Esther Imperlini, Ersilia Nigro, Concetta Montagnese, Aurora Daniele, Stefania Orrù, Pasqualina Buono

**Affiliations:** 1 Dipartimento di Scienze Motorie e del Benessere, Università di Napoli “Parthenope”, via Medina 40, Napoli 80133, Italy; E-Mails: annamaria.mancini@uniparthenope.it (A.M.); orru@uniparthenope.it (S.O.); 2 CEINGE Biotecnologie Avanzate s.c.a r.l., via G. Salvatore 486, Napoli 80145, Italy; E-Mails: imperlini@ceinge.unina.it (E.I.); nigro@ceinge.unina.it (E.N.); montagnese@ceinge.unina.it (C.M.); aurora.daniele@unina2.it (A.D.); 3 Dipartimento di Scienze e Tecnologie Ambientali Biologiche Farmaceutiche, Seconda Università degli Studi di Napoli, via G. Vivaldi 42, Caserta 81100, Italy

**Keywords:** palm oil, palmitic acid, obesity, T2DM, cardiovascular diseases, cancer, SFA, TAG, PUFA, MUFA

## Abstract

A growing body of evidence highlights the close association between nutrition and human health. Fat is an essential macronutrient, and vegetable oils, such as palm oil, are widely used in the food industry and highly represented in the human diet. Palmitic acid, a saturated fatty acid, is the principal constituent of refined palm oil. In the last few decades, controversial studies have reported potential unhealthy effects of palm oil due to the high palmitic acid content. In this review we provide a concise and comprehensive update on the functional role of palm oil and palmitic acid in the development of obesity, type 2 diabetes mellitus, cardiovascular diseases and cancer. The atherogenic potential of palmitic acid and its stereospecific position in triacylglycerols are also discussed.

## 1. Introduction

Over the last few decades there has been growing public concern about the significant interplay between health and nutrition. Fat is an essential macronutrient of the human diet and vegetable oils represent a more highly consumed fat. The effects of high-fat diet, mainly in saturated fatty acids (SFA), have been the focus of several dietary guidelines targeting the reduction of cardiovascular diseases (CVD) [1,2], obesity-related diseases and, recently, cancer prevention [3].

The increasing demand for vegetable oils is a worldwide phenomenon and palm oil (PO) contributes significantly to the global supply of edible oils. PO is entirely GMO-free and produces up to 10 times more oil per unit area than other oilseed crops. In 2012 PO accounted for 32% of global fats and oils production and it has overtaken soybean oil as the most important vegetable oil in the world [4].

The palm tree (*Elais guineensis*) is an ancient tropical plant native to many West African countries, where local populations traditionally use its oil for cooking and other purposes. Large scale palm crops are found across the tropical regions, and Malaysia and Indonesia are the leading producers of PO, accounting for the 86% of global production; other PO producing countries are Nigeria, Thailand, Colombia, Papa Guinea, Cote d’Ivoire, India and Brazil.

Two different types of oil are extracted from palm fruit: palm kernel oil (PKO), from the seeds and PO, from the mesocarp. The edible oil contained in the mesocarp of palm fruit can be extracted using different methods, with the most common known as wet or dry processes [4].

Crude palm oil (CPO, known also as red palm oil, RPO), extracted either by wet or dry processes, contains both healthy beneficial compounds, such as triacylglycerols (TAGs), vitamin E, carotenoids, phytosterols, as well as impurities, such as phospholipids, free fatty acids (FFAs), gums, and lipid oxidation products; the latter can be removed by means of refining processes [5]. Hence, the CPO is purified by centrifugation and drying; the dried oil is then cooled and stored in appropriate containers [6].

CPO represents the richest natural source of carotenoids (500–700 ppm), tocopherols and tocotrienols (600–1200 ppm), all contributing to its stability and nutritional properties [4,5,7,8,9]. Their antioxidant properties, exerted mainly against reactive oxygen species (ROS), play a role in aging, in CVD and in cancer prevention [7,10,11]. Furthermore, tocotrienols have been reported to be natural inhibitors of cholesterol synthesis [7].

Despite the good quality of CPO, food manufacturing industries require PO with a bland and light color, making refinery a mandatory step. These steps can be carried out via chemical (alkaline or acid treatment) [12,13] or physical methods (steam refining, inert gas stripping, molecular distillation, membrane refining, *etc.*) [13,14]. Afterwards, bleaching and deodorizing steps follow, along with other specific treatments depending on the first refining process applied. For example, after alkali treatment, the impurities are removed by centrifugation as gums; volatile products are then removed through stream distillation at higher temperature and reduced pressure. The oil obtained after the refining processes is colorless, bland and stable. On the other hand the alkali treatment produces losses in neutral TAGs and it is a more expensive process with a high impact on the environment. For these reasons, physical refining protocols, based on different volatility of FFAs and TAGs, high-temperature and low-pressure methods, are currently used [13,14], even if these procedures lead to an evident loss of tocopherols and tocotrienols and also produce oxidative damages [13]. Moreover, PO refined by physical treatments shows lower storage stability and require an accurate procedure in order to eliminate phosphorus during the degumming procedure, to avoid the presence of phosphoric acid in the final product that would affect the heat bleaching process [13].

PO with low amounts of free fatty acids (FFAs), low impurity content and good bleaching is considered of high quality and used in the edible oil industry; conversely, low-quality oils are used in non-edible industry such as biofuel, candles, cosmetics and soap production [15]. So-called high-quality PO is constituted of more than 95% neutral triacylglycerols (TAGs, or triglycerides) and less than 0.5% FFAs [13,14].

PO and PKO have different physical and chemical properties depending on their intended applications [4,10]. As shown in Table 1, PKO contains 85% SFAs, mainly lauric and myristic acids, while PO contains 50% SFAs, mostly palmitic acid (PA, 44%) and lower amounts of stearic acid (5%), 40% monounsaturated fatty acids (MUFAs), mostly oleic acid, and 10% polyunsaturated fatty acids (PUFAs), mostly linoleic acids [5,7,16]. PA is the main SFA naturally occurring in animal fats and vegetable oil, as well as the main component of human milk fat [17].

**Table 1:** Fatty acid composition of palm oil and palm kernel oil.

Fatty Acid	Palm Oil	Palm Kernel Oil
Caproic acid (6:0)	-	0.2
Caprylic acid (8:0)	-	3.3
Capric acid (10:0)	-	3.5
Lauric acid (12:0)	0.2	47.8
Myristic acid (14:0)	1.1	16.3
Palmitic acid (16:0)	44.0	8.5
Stearic acid (18:0)	4.5	2.4
Oleic acid (18:1)	39.2	15.4
Linoleic acid (18:2)	10.1	2.4
Linolenic acid (18:3)	0.4	-
Arachidic acid (20:0)	0.1	0.1
Total SFAs	49.9	82.1
Total MUFAs	39.2	15.4
Total PUFAs	10.5	2.4

Data partially obtained and adapted from [7]. -: absent.

In the last few decades, PO’s application in food industries has exponentially grown for the texture, the fragrance and the neutral taste it guarantees in the finished products. Its two major fractions are the low-melting liquid fraction (known as palm olein, 65%–75%) and the high-melting solid fraction (known as palm stearin, 30%–35%). The different palm oil fractions are then differently used in food industry: palm olein is used in cooking oil for frying (because of its high smoke point, 230 °C) and margarines; palm stearine in food applications such as shortenings and hydrogenated oils used as butter substitutes in India; double-fractioned palm olein is then used in mayonnaise preparation [4]. PO is generally found in: baked goods, candies, cakes, cheese analogs, chips, chocolate, confectionary fats, cookies, cooking oil, crackers, doughnuts, frozen meals (pancakes, pies, pizza, potatoes), ice cream, industrial frying fats, instant noodles and oatmeals, margarines, microwave popcorn, non-dairy creamers, peanut butter, salad dressings, snacks, soups, supplements/vitamins, vegetable ghee.

**Figure 1:**
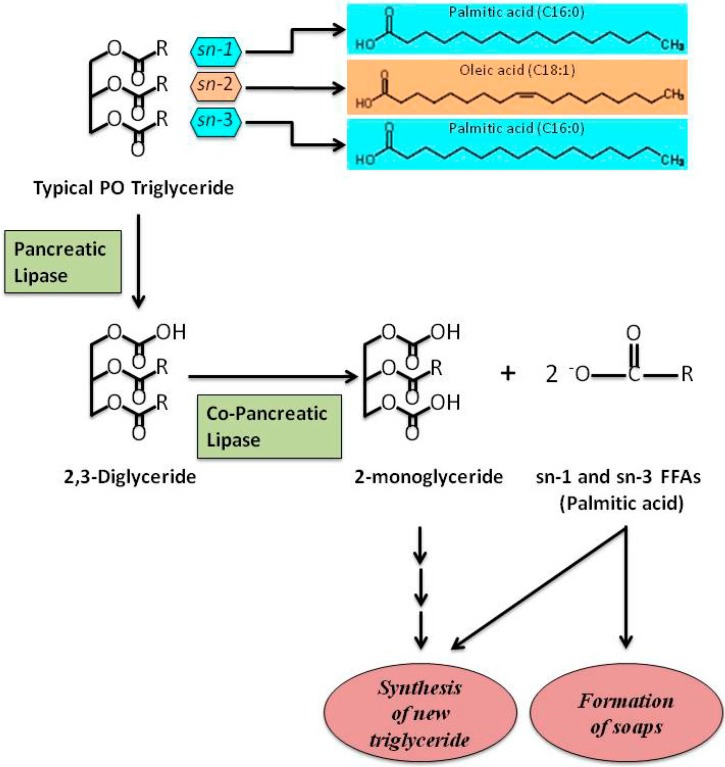
A schematic representation showing the hydrolysis of typical PO triglyceride. Palmitic acid, the main SFA in PO, is situated at SN-1 and SN-3 positions of TAGs. The other main FA in PO, oleic acid, is situated at SN-2 position. After digestion, FFAs are involved in the re-synthesis of new triglycerides and/or formation of Ca^2+^ or Mg^2+^ soaps. Free fatty acids, FFAs; palm oil, PO; saturated fatty acid, SFA; triglycerides, TAGs.

Despite some authors suggesting that palm olein, containing oleic acid at 48%, could be a substitute for olive oil in healthy normo-cholesterolaemic human diet [5], debates related to PO’s potential unhealthy effects (mainly due to the high PA content) are ongoing [18,19,20,21]. However, PO, like all vegetable oils, is cholesterol-free and FAs are mainly structured as TAGs. TAGs contained in PO are mainly constituted of oleic acid, predominantly located at the SN-2 position, and of PA, mainly located at the SN-1 and SN-3 positions (Figure 1). TAGs contained in animal fat are different in that PA or stearic acid are often found at the SN-2 position [5,22].

## 2. Palm Oil and Palmitic Acid: Role in Obesity and T2DM

Obesity is a metabolic disease characterized by an excess of white adipose tissue (WAT) resulting from an excess of energy stored in adipocytes as TAGs, responsible for adipocyte hypertrophy and hyperplasia [23]. In recent years, obesity has assumed epidemic proportions: about 1.9 billion adults worldwide and 42 million children below 5 years are obese [24]; morbidity and mortality associated with obesity-related diseases, such as type 2 diabetes mellitus (T2DM), CVD, hyperlipidemia and hypertension, have increased exponentially [25].

WAT produces adipokines that are responsible of chronic inflammation processes and are associated with obesity-related metabolic diseases [26]. Adipose tissue from obese mice contains elevated SFA depots that activate Toll-like receptor 4 (TLR4)-mediated inflammatory signaling [27,28,29].

SFAs also stimulate pro-inflammatory mechanisms through reactive oxygen species (ROS) in a TLR-independent manner. ROS regulate the activation of mature interleukin-1β (IL-1β) from its inactive precursor pro-IL-1β [30,31]. As a consequence, IL-1β down-regulates the insulin-signaling in insulin target cells, providing a possible SFA-mediated inflammatory response resulting in insulin resistance [32,33].

More recently, a link was suggested among high-fat diet, inflammation and pro-inflammatory products in plasma originated in the gut by the death of Gram-negative intestinal microbiota, namely lipopolysaccharides (LPSs) or endotoxins [34,35]. LPSs, bound to LPS-binding proteins, trigger inflammation via the TLR4 pathway and induce secretion in the plasma of pro-inflammatory cytokines, such as IL-6 [36]. Moreover, several studies showed that metabolic endotoxemia could be generated by gut LPS absorption during digestion of a high-lipid load [37,38]. A direct effect of PA on increased secretion of IL-6 in 3T3-L1 adipocytes, compared to myristic, linoleic or α-linolenic acids, was reported by Laugerette and co-authors [39]; they also demonstrated the cooperative effect of PA and LPS co-treatment in these cells, confirming the role of LPS in modulating inflammation through different dietary fat contents. Similarly, the same authors found that PO-enriched diet induced higher amounts of inflammatory markers in plasma (IL-6) and in WAT (IL-1β, TLR4 and CD14) compared to other fat-based diets in mice [39].

**Figure 2:**
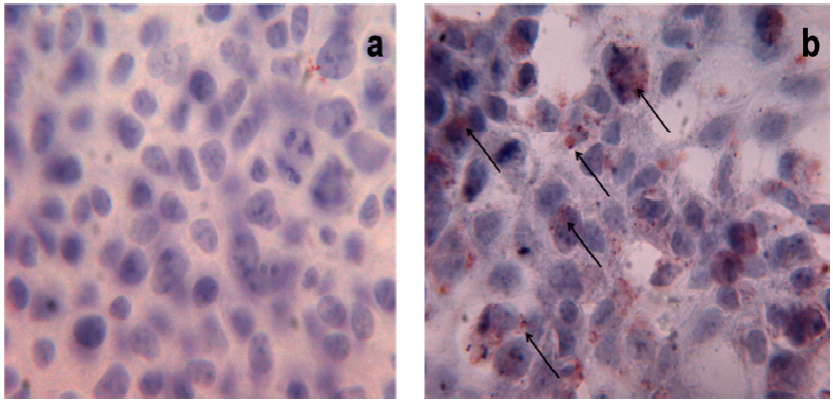
Triglyceride storage in HEK293 cells. Oil Red O staining of untreated (**a**) and treated (**b**) HEK293 cells with PA 1 mM. Red points indicate triglyceride depots; ×40 magnification.

There is growing evidence linking obesity to changes in gut microbiota [40,41]. PO-rich diet determines weight-gain and hepatic lipid accumulation compared to unsaturated fat diet (olive or safflower oil) in C57BL/6J mice, together with a reduced microbial diversity in the intestine [42]; these observations support the hypothesis that an excessive consumption of PO in the diet triggers changes in gut microbiota components and determines lipid accumulation. Triglyceride-droplet accumulation mediated by high doses of PA (500 μM and 1 mM) has been also described in human HEK293 cells (Figure 2) [43]; interestingly, the authors highlighted the role of uncoupling protein-3 (UCP3) in the metabolism of long-chain fatty acids, such as PA, in these cells.

The effects of PO, and of its main constituent PA, have also been investigated on other organs and tissues, such as the central nervous system (CNS).

The effects of intra-cerebroventricular (icv) injection of PA on hypothalamic leptin signaling, inflammatory marker secretion and hepatic energy metabolism were recently analysed in C57BL/6J male mice [44]. The authors reported that high-doses of PA induce pro-inflammatory responses and leptin resistance, similarly to obesogenic-diets; however, the key role of PA in the hypothalamus in response to high-fat diets has not been fully characterized yet.

Another interesting aspect is the relation between diet, with different content in lipids consumed during pregnancy and lactation, and development of obesity in adult life. The fetal nutritional environment is involved in obesity and/or obesity-related diseases in later life [45,46]. Several studies carried out on animal models indicated that, during lactation, the consumption of normolipidemic diets, rich in SFAs derived from PO and/or in partially hydrogenated fats (PHF) induces fat retention in the young offspring [47]. In fact, maternal intake of PO and inter-esterified fat (IF) present in processed foods predisposes the offspring to the development of obesity in adult life [48].

In obesity, an altered production of pro-inflammatory cytokines, such as TNF-α, IL-6, leptin and adiponectin, affects both secretion and efficiency of insulin action, hallmarks of T2DM, along with progressive failure of β cells [49,50,51]. In fact, obesity and T2DM are strongly interconnected and their overlap leads to predisposition to a large number of metabolic diseases, including metabolic syndrome [52,53].

Oxidative stress and ROS overproduction are significantly increased in T2DM, promoting endothelial dysfunction, a condition more prone to the pathogenesis of hypertension and CVD in diabetic patients. Recently, T2DM was also linked to immune system activation and inflammation [54].

The role of PO consumption in T2DM is controversial in that, it is difficult to exactly distinguish between the effects of PO and/or other fat components of the diet in T2DM risk. Some evidence indicates that diet supplemented with PO impairs glucose tolerance in mice [55]; this finding may be ascribed to the reduction in insulin-sensitivity induced by PO-enriched diets and to the corresponding increase of serum TAGs; in fact, elevated serum TAGs are associated with insulin-resistance [56]. Furthermore, several studies, involving animals and humans, have shown that fatty acid composition of diet affects muscle membrane phospholipid dynamics (such as fluidity) and the ligand/receptor recognition process (such as insulin/insulin receptor) [57]. On rat epididymal fat cells, PO treatment resulted in a lower rate of insulin-stimulated glucose uptake due to reduced insulin binding to the cells compared to sunflower oil treatment [58].

Studies conducted on humans yielded conflicting results: four weeks of PO treatment in 30 patients affected by T2DM had no significant effects on plasma glucose concentration [59]; on the contrary, Rosqvist and co-authors demonstrated in 39 T2DM patients that an excessive consumption of SFAs (among which PA) favors both liver and visceral fat accumulation with detrimental role in T2DM development and progression [60]. Also the largest study on T2DM patients, the EPIC-InterAct case-cohort study, was not indicative. In fact, the study, including 12.403 Caucasian people with incident T2DM, reported that different SFAs (myristic, palmitic, and stearic acid) have different effects on T2DM incidence. Interestingly, the authors underlined that PA can also be synthesized endogenously through *de-novo* lipogenesis, a metabolic pathway stimulated by increased intake of carbohydrates and alcohol; these evidences introduce a further complexity level in this puzzle making very difficult to distinguish between endogeneous and exogeneous PA effects on T2DM onset risk [61]. Furthermore, *in vitro* studies demonstrated that PA directly impairs insulin signaling in cultured rat hepatocytes and myotubes as well as pancreatic cells vitality and insulin secretion [62]. Three main mechanisms have been suggested: (1) reduction of viability and induction of apoptosis through ER stress. In rat insulinoma cells, PA provokes toxicity through lipotoxic stress in ER; this mechanism, exerted by different SFAs, has been described in various cell types [50,63,64,65]. In addition, SFAs can directly interact with ER membrane bilayer disturbing ER morphology and function [57]; (2) inhibition of insulin-stimulated phosphorylation event. PA markedly impairs phosphorylation and, therefore, activation of insulin receptor, insulin receptor substrate-1, and Akt in several cell types, contributing to the onset of insulin-resistance [66]; (3) ubiquitination of the key insulin-signaling molecules. PA facilitates the ubiquitination and, hence, elicits proteasome degradation of insulin receptor, insulin receptor substrate-1, and Akt [67].

## 3. Palm Oil and Palmitic Acid: Role in CVD

CVD represent the principal cause of death worldwide. Main serum/plasma CVD-related biomarkers are TC, LDL-C, high-density lipoprotein cholesterol (HDL-C), TAGs and very low-density lipoprotein cholesterol (VLDL-C). Also apolipoprotein A-I (apo A-I) and B (apo B) reflect variations in HDL-C and LDL-C, respectively [68,69]. In particular, low apo A-I and high apo B levels are associated with increased CVD risk and the apoB/apo A-I ratio is considered a good CVD predictor factor [70].

The link between PO consumption and CVD risk refers to PA content in PO, which accounts for 44% of total fat (Table 1). Since 1950 Toshima’s group demonstrated that high contents of SFAs in diets are powerful predictors of heart diseases, the authors provided a specific equation to predict serum cholesterol levels starting from SFA intake [71,72,73,74]. Successively, the “lipid theory” stated that an excessive SFA consumption is responsible for hypercholesterolemia, a condition that causes predisposition to a higher CVD risk: an increase of 20 mg/mL in total serum cholesterol determines a 12% higher CVD risk [75]. At the same time, Mensink and co-authors [76,77] demonstrated that the most favorable lipid profile is obtained when MUFAs or PUFAs replace SFAs.

In a multi-country analysis Chen and co-authors [78] evaluated the effects of PO consumption on CVD mortality-risk due to ischemic heart disease (IHD) and stroke. Twenty-three countries were analyzed and divided into historically high-income countries (HICs) and developing countries (DCs). The authors reported that every additional kg of PO consumed per-capita annually determined higher IHD mortality rate in DCs respect to HICs. The same trend was also observed in stroke deaths, but in this case, data were not statistically significant, suggesting that serum LDL-C is not strictly linked to hypertension.

Nevertheless, it is now emerging that both favorable and unfavorable changes in CVD biomarkers are observed when PO constitutes the main dietary SFA intake [19,20]. In studies where PO was compared to soybean oil (a vegetable oil with more PUFAs and less SFAs) [19,79], to olive oil (rich in oleic acid, a MUFA) [19,80], to sunflower oil (rich in oleic acid and PUFA) [19,80] and to canola oil (rich in MUFAs) [19,81], no substantial differences in the lipid serum profile were observed. In some cases, changes in cholesterol fractions, not affecting TC/HDL-C or LDL-C/HDL-C ratios, were registered. Furthermore, other studies failed to unequivocally demonstrate the association of PA consumption and CVD risk increase, particularly in normocholesterolemic subjects assuming the recommended PUFA intake [82,83]. Finally, SFA intake, associated with PO consumption, did not significantly affect the apoB/apoA-I ratio [84,85,86].

In addition, it is well known that the number of carbons in the FA chain, the degree of saturation and the stereospecific positioning of FAs in TAG structures highly influence FA absorption, thus affecting their metabolism and their role in CVD [10,87,88]. In fact, CVD biomarkers (TC, LDL-C, HDL-C and VLDL-C) worsen when main dietary SFAs are characterized by shorter carbon chains: lauric (C12:0) and myristic acids (C14:0) increase all the cholesterol fractions (favorable and unfavorable) more than PA (C16:0); this latter increases all the cholesterol fractions (favorable and unfavorable) more than stearic acid (C18:0) [89,90]. Furthermore, the atherogenicity of a specific TAG is associated to the degree of saturation of FA located at SN-2 position [91,92,93,94,95]: in fact, it has been shown that higher percentages of PA at SN-2 position are related to the most atherogenic profiles. Accordingly, Kritchevsky and co-authors reported that lard is highly atherogenic because almost all PA (21.4%) is at SN-2 position in the TAGs. Interestingly, when a randomization process is applied to modify the TAG structure, the presence of PA at SN-2 position is reduced by about 67% and, consequently, the atherogenic potential decreases to the half [93]. Likely, tallow has a similar content in PA (24.8%) with a smaller fraction (3.8%) being at SN-2 position; following randomization, the amount of PA at SN-2 position increases by about 124%, and the atherogenic potential raises [93]. Comparing different animal fats and vegetable oils, the percentage of PA in SN-2 position of corresponding TAGs is 66% in animal butter, 58% in human milk, 34% in bovine milk, 4.4% in PO and 0.3% in olive oil [88]. In fact, as shown in Figure 1, the typical TAGs of PO are symmetrical with PA bearing the SN-1 and the SN-3 positions and oleic acid the SN-2 position. Long chain SFAs impair absorption rates from the intestinal lumen when they occupy SN-1/SN-3 position of glycerol backbone; following lipase action, they are converted in FFAs and tend to form insoluble soaps, excreted in faeces [96], instead of being transformed into bile salt micelles or vesicles [87]. These evidences are associated with a reduced atherogenicity and support the concept that PO carries a low CVD risk within balanced diets [10].

The atherogenic effects of PO were also compared to CPO and to reconstituted CPO (PO added with carotenoids, tocotrienols and tocopherols) [95] in rabbits fed with semipurified diets containing 0.1% cholesterol and 13% test oil (PO, CPO, reconstituted CPO) for 90 days. At the end of the treatments, serum cholesterol levels were comparable among rabbit groups, whereas reconstituted CPO determined a higher increase of TAGs. Authors hypothesized that the fine structure of native CPO is different from the reconstituted CPO, and that the absorption of carotenoids and vitamin E in native CPO could be different, leading to the idea that natural products cannot be easily reproduced by simply adding and mixing specific components.

## 4. Palm Oil and Palmitic Acid: Role in Cancer

There is now considerable evidence that, in addition to the known risk factors, dietary fat intake plays an important role in determining cancer risk [97,98,99]. For decades, epidemiological studies indicated a positive association between total fat intake and risk of breast, colorectal and prostate cancers. Starting from this evidence, it has been postulated that a high-fat diet (>25% FAT) induces a major risk of cancer development compared to low-fat diet (<20% FAT). The dietary fat hypothesis is supported by several meta-analysis studies obtained from data collected up to nowadays [100,101,102]. Retrospective case-control investigations also report a relationship between increased fat intake and higher cancer risk with conflicting results.

In a recent prospective study in postmenopausal women, the authors found positive associations between total MUFA intake or PA and stearic acid intake and breast cancer incidence [97]. In agreement with these data, a previous meta-analysis on prospective studies revealed that high levels of PA were associated with an 89% increased risk of postmenopausal breast cancer [100]. Although this study supports the hypothesis of breast cancer risk associated with fat consumption, most of the prospective studies failed to confirm this hypothesis, reporting inverse associations [103,104] or no associations [105].

To our knowledge, only a few studies reported significant increased risks of breast cancer associated with MUFA consumption [106].

Interestingly, a national prospective case-control in Scotland showed that PA, together with total MUFAs and others SFAs, were positively associated with colorectal cancer risk in a dose-dependent manner; on the other hand, these effects were abolished after adjusting for potential confounding factors such as family history of cancer, total energy and/or fiber intake, drugs, smoking, body mass index, and physical activity [107].

As for prostatic cancer, epidemiological studies lack to find a consistent association between FA intake and cancer risk. However, a case control analysis conducted on plasma FAs revealed a positive association between PA consumption and risk of localized and low-grade prostate cancer [108]. Furthermore, a prospective cohort study in Japan showed that PA increased the risk of prostate cancer in a dose-dependent manner [109], whereas higher intakes of dietary MUFA as well as moderate concentrations of plasma PA were inversely related to prostate cancer [99].

The conflicting results of these studies may be due to (i) sample size that, in same case, was not large enough to observe statistical significant positive associations; (ii) use of different biomarkers of FA intake (blood or adipose tissue) with different time-frame exposure: in fact, FA profile of adipose tissue reflects the relative intake over 2 years, whereas FA composition of blood indicates recent intake; (iii) the heterogeneity of study populations that display different characteristics (*i.e.*, age, hormonal status, body mass index, physical activity), additional cancer risk factors (obesity, weight loss, smoking, drugs and alcohol) and other pathological conditions (presence or not of cancer) affecting lipid metabolism and, as a consequence, FA composition.

Different effects of SFAs, such as PA, and MUFAs, have been reported on cell proliferation and tumor development also in *in vitro* and animal studies [110,111]. The conflicting results may be due to a combined administration in animals of different FAs. However, such results raised new questions about the key role played by the type of FAs (saturated, monounsaturated, and polyunsaturated) besides to their amounts.

It has been reported that PUFAs of the linoleic group (*n*-6 PUFAs) stimulate breast, colorectal and prostate cancer development and progression [112,113], while the PUFAs of the linolenic group (*n*-3 PUFAs), especially those from marine origin (eicosapentaenoic acid and docosahexaenoic acid), inhibited tumor growth [114].

Interestingly, Rossini *et al.* demonstrated in mice that a FA-free diet reduces mammary tumor incidence but not tumor growth rate [111]. Because humans are not able to comply with a FA-free diet, Rossini’s study suggests that a low-fat diet might play a protective role in tumorigenesis but no effects should be expected on already established tumor masses.

Similarly to breast cancer, positive associations with dietary PA, MUFA and n-6 PUFA contents have been reported for rectal, but not for colon cancer; *n*-3 PUFAs were, instead, inversely associated with cancer risk [98].

All the reported studies carried out on animal models or humans support the hypothesis that, in addition to the total amount and the type of FAs, also their ratios in the diet may influence carcinogenesis. Kuriki and co-authors reported that the colorectal cancer risk was positively associated not only with erythrocyte membrane content of PA but also with ratio SFAs/PUFAs [115].

On the contrary, Shannon and co-authors reported a significant direct association among both PA and palmitoleic acid and breast cancer risk when the two SFAs were analyzed independently; instead, when their ratio (PA *vs.* palmitoleic acid) was considered, a significant inverse association with breast cancer risk was observed [116]. This latter finding may be due to a lower concentration of palmitoleic acid that is produced through desaturation of PA by δ-9 desaturase; PA is, in turn, the primary end product of fatty acid synthase (FAS) reaction.

Although there is now evidence that individual FAs, such as PA, and their ratios are more reflective of lipid metabolism in comparison to the total fat dietary intake, little is known about the potential mechanisms by which these SFAs may trigger carcinogenesis.

At molecular level, FA intake could affect cancer development and progression through the modifications and/or alterations of: (i) hormonal status; (ii) composition of cell membrane and (iii) cell signaling transduction pathways.

As previously reviewed, adiposity associate to FA intake can stimulate *de novo* synthesis of hormones, such as estrogens, whose production induces cell proliferation, thus determining a higher cancer risk [117].

Moreover, the FA intake could affect the SFAs/MUFAs ratio in the membrane phospholipid, thus altering many membrane-associated functions.

It has also been proposed that FA intake could have an immunosuppressor effect and determine a cancer risk by altering cell membranes of the immune system [118]. Also protein composition of the cell membrane could be affected by dietary intake of specific FAs with consequences on cell to cell interactions and cell’s response to growth factors [119]. We cannot exclude possible membrane protein modifications by palmitoylation and myristoylation leading to alterations of localization and function of key proteins for tumor suppression.

*In vitro* and *in vivo* studies suggested that specific FAs could promote cell invasiveness or metastasis [120,121]. However, only confusing results have been obtained on the action of selected FAs in cell signaling transduction pathways, in particular in cell proliferation and apoptosis.

In this context, Hardy and co-authors reported the opposite effects on breast cancer cell proliferation of the two most abundant circulating FAs, PA (saturated) and oleic acid (unsaturated). In particular, the authors reported that PA induces apoptosis and oleic acid promotes cell proliferation and prevents the proapoptotic effect induced by PA [110]. The action of both saturated and unsaturated FAs seems to be mediated by PI3-K, but the potential mechanism remains uncharacterized, as well as the pro-apoptotic mechanism of PA.

Moreover, the inhibition of FAS activity and its gene expression, mediated by PA, could be further investigated as a potential mechanism of PA involvement in cancer.

Interestingly, FAS, a key enzyme of FA metabolism, is overexpressed in many human cancers [122] including not only breast and colorectal cancer, but also the non-small-cell lung cancer, representing a leading cause of cancer mortality in the world [123]. Therefore, the emerging evidence that FAS is a potential prognostic tumor marker and a target for anticancer drug development [124] strengthens the hypothesis that specific FAs, such as PA, may strictly be involved in the regulation of tumor growth.

### PA and Cell Toxicity

Intracellular FAs are essential components of cells and serve mainly as an energy source. Besides their well-known cellular functions, there is now emerging evidence on FA’s roles in cells that could trigger adverse unhealthy effects when FA homeostasis is compromised [125]. Cellular lipid droplets and long-chain (LC) FAs cannot penetrate the lipid bilayer of mitochondria, so the cells digest and use lipid droplets through an autophagic degradative pathway [126,127]. Moreover, an excessive accumulation of cellular FFAs may cause lipotoxicity in non-adipose tissues and such a phenomenon is considered an hallmark for metabolic diseases. Until now the mechanisms leading to cell-toxicity have not been completely understood. In general, LC SFAs, such as PA, are directed to the mitochondria for oxidation and/or to endoplasmic reticulum (ER) for complex lipid synthesis; on the contrary, SFA excess may provide modification in phospholipid composition of ER membrane, compromising its structure and integrity [128]. LCFA treatment induces apoptosis in different established or primary cell types: in fact, PA overload induce apoptotic cell death by ER stress [129,130]; moreover elevated concentrations of PA determine apoptosis and autophagy through mitochondrial dysfunction and ER stress, mediated by oxidative stress increase, in hepatocytes [131], pancreatic beta cells [132] and muscle cells [133,134,135]. FFAs are oxidized by the β-oxidation process in mitochondria, the most important source of reactive oxygen species (ROS) [136]. As quoted above, mitochondrial ROS generation induced by PA, in subjects with a high-fat/sucrose diet or obese, desensitizes the insulin signaling pathway via JNK (through TLR4) and impairs IRS-2 phosphorylation in hepatic cell lines; conversely, treatment with anti-oxidants prevent the insulin-resistance and reduce the ROS generation [137].

The development of lipotoxicity has been also related to oxidative stress in human cells and in animal models [138]. Moreover, DNA and protein oxidation products, pro-oxidant, are elevated in serum and in the β-cells derived from T2DM subjects with hyperlipidemia [139,140]. Among the different mechanisms responsible for lipotoxicity and oxidative stress in different cells, there is the increased production of ceramide after FFA overload in β-cells. Ceramide is a second messenger involved in the apoptosis and the inhibition of *de novo* ceramide synthesis prevents apoptosis and lipotoxicity in β-cells, but not in other cells, suggesting that lipotoxicity mechanisms are tissue-specific. Ceramide can in turn activate NADPH oxidase, and disrupt mitochondrial respiration chain promoting the release of cytochrome c [141].

The effects of SFAs on mitochondrial dysfunction have been widely investigated in human hepatoma cell lines, demonstrating that SFA treatments decrease the activity of protein enzymatic complexes of oxidative phosphorylation (OXPHOS) with reduction of ATP/ADP ratio; this effect is rescued by anti-oxidants suggesting that this phenomenon is due to the increase of ROS production in treated cells. It seems that PA induces both a reduced synthesis and an accelerated degradation of OXPHOS complexes: both processes are ascribed to nitrosative stress due to 3-tyrosine nitration of mitochondrial proteins [142]. Furthermore, PA, can exert its oxidative stress effects on CYP2E1 cytochrome and the treatment with CYP2E1 inhibitors prevents the effects of PA on OXPHOS complexes [143]. Finally, endothelial cells exposed to chronic, elevated concentration of PA trigger a lipotoxic response leading to necrotic cell death. In fact, PA induces programmed necrotic death (necroptosis) in endothelial cells through Ca-dependent autophagy activation [144]. Hence, autophagic clearance represents a novel molecular mechanism of PA-induced lipotoxicity.

## 5. Conclusions

Palm oil represents the most widely used vegetable oil in the world. PO is found in supermarket products ranging from margarine, cereals, sweets and backed goods. In the last decades many studies focused on the potential unhealthy effects of PO in the diet, due to its high PA content, with controversial results. In animal models diet supplemented with PO induces impaired glucose tolerance that can be ascribed to the reduction in insulin-sensitivity. Studies conducted on humans showed that different SFAs present in the diet have different effects on T2DM incidence.

Regarding the effects mediated by PO-rich diets on CVD risk increase in human, conflicting results have been reported. The main criticisms can be summarized as follow: (a) the extreme qualitative and quantitative heterogeneity of FA contents in the diets; (b) the differences in selection criteria used for intervention and control groups enrollments; (c) the wide-range of investigated ages and (d) the poor attention paid towards other dietary components that can confound the direct FA effects on blood lipid markers. Recently, an increasing amount of evidence has highlighted the negative effects of PA excess on mitochondrial function mediated by oxidative stress, an effect known as lipotoxicity. However, until now no clear evidence has been provided to unequivocally demonstrate the association of PA consumption and CVD risk-increase, particularly in normocholesterolemic subjects assuming the recommended PUFA intake; in addition, the percentage of PA at SN-2 position in TAGs is reduced in PO compared to animal fat, supporting the hypothesis of the low atherogenic power of PO when assumed in balanced diets and that adverse effects could be due to a dose-response relationship. Such considerations suggest that more rigorous investigations are needed to define the net advantages and disadvantages induced by PO consumption on CVD.

Also the association between PA consumption and cancer gives rise controversial results. This may be ascribed to the heterogeneity of study population, to the difficulty of taking into consideration additional risk factors, and other pathological conditions affecting lipid metabolism. However, some studies found that low-fat diet plays a protective role only in the tumorigenesis process with no effect on established tumor mass. Furthermore, the type of FAs (*n*-6 MUFA *vs. n*-3 PUFA) present in the diet also influences the carcinogenesis process. In conclusion, specific FAs as PA may be strictly involved in regulation of tumor growth.

**Figure 3:**
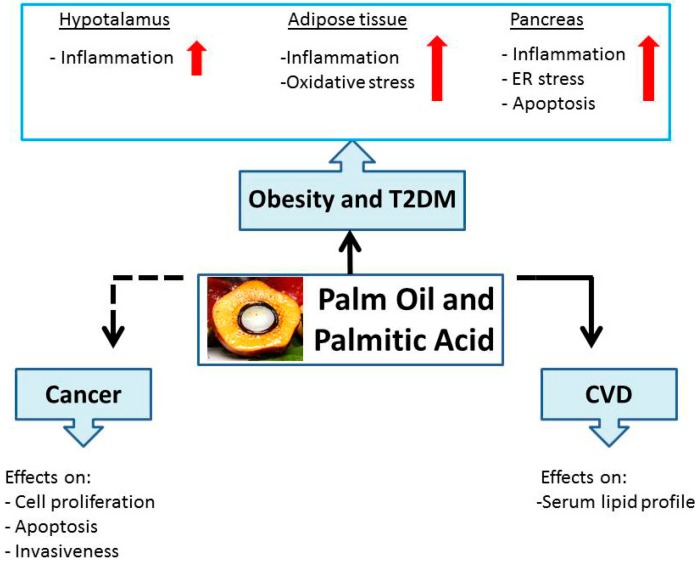
Schematic representation of palm oil and palmitic acid effects on human health.

A schematic diagram representative of PO and PA effects on health, discussed in this review, is summarized in Figure 3.
